# Primary cardiac sarcomas: A multi‐national retrospective review

**DOI:** 10.1002/cam4.1897

**Published:** 2018-12-21

**Authors:** Tom Wei‐Wu Chen, Herbert H. Loong, Amirrtha Srikanthan, Alona Zer, Reeta Barua, Jagdish Butany, Robert J. Cusimano, Yun‐Chieh Liang, Chin‐Hao Chang, Zaza Iakobishvili, Albiruni R. Abdul Razak, Jeremy Lewin

**Affiliations:** ^1^ Department of Oncology National Taiwan University Hospital Taipei Taiwan; ^2^ Graduate Institute of Oncology National Taiwan University College of Medicine Taipei Taiwan; ^3^ National Taiwan University Cancer Center Taipei Taiwan; ^4^ Department of Clinical Oncology, State Key Laboratory of Oncology in South China The Chinese University of Hong Kong Hong Kong SAR; ^5^ BC Cancer Vancouver British Columbia Canada; ^6^ Rabin Medical Center Tel Aviv University Tel Aviv Israel; ^7^ Department of Medicine University of Toronto Toronto Ontario Canada; ^8^ Division of Pathology University Health Network, University of Toronto Toronto Ontario Canada; ^9^ Division of Cardiovascular Surgery, Department of Surgery University Health Network, University of Toronto Toronto Ontario Canada; ^10^ Clinical Trial Center National Taiwan University Hospital Taipei Taiwan; ^11^ Department of Medical Research National Taiwan University Hospital Taipei Taiwan; ^12^ NHMRC Clinical Trials Centre University of Sydney Camperdown New South Wales Australia; ^13^ Sarcoma Department Mount Sinai Hospital Toronto Ontario Canada; ^14^ Department of Medical Oncology and Hematology Princess Margaret Cancer Centre Toronto Ontario Canada; ^15^ Present address: Peter MacCallum Cancer Centre Melbourne Victoria Australia

**Keywords:** primary cardiac sarcoma, radiotherapy, sarcoma chemotherapy, surgery

## Abstract

**Background:**

Primary cardiac sarcoma (PCS) is a rare but often fatal disease. The current study aimed to analyze the impact of baseline demographics, local and systemic therapies in a contemporary cohort.

**Methods:**

Clinical records of PCS across six institutions in three continents were reviewed. Kaplan‐Meier method was used to estimate survival. Cox proportional hazard model was used to determine variables impacting progression‐free survival (PFS) or overall survival (OS).

**Results:**

Sixty‐one patients with PCS (1996‐2016) were identified. The median age at diagnosis was 46 (range 18‐79); 36% (n = 22) presented with metastatic disease. The most common histology was angiosarcoma (n = 24, 39%). A total of 46 patients received surgery (75%) but only 5 (8%) patients achieved R0 resection. Multi‐modality treatment to the primary tumor was given to 28 patients (46%; localized disease 23/39 (59%); metastatic disease 5/22 (23%)). The median OS for the entire cohort was 17.5 months (95% CI 9.5‐20.6), with seven (11%) patients surviving longer than 36 months. On multi‐variate analysis, age <65 (*P* = 0.01) was the only significant favorable prognostic factor. For first‐line palliative chemotherapy, the median PFS was 4.4 months (95% CI 2.9‐7.7 months). The best response for first‐line chemotherapy was 32% (CR = 1, PR = 9). No significant improvement in OS was identified in patients presenting throughout the 20‐year period of this review.

**Conclusion:**

Younger age at diagnosis was associated with improved outcome although the prognosis of PCS remains poor. Given the lack of improvement in survival, further dedicated research is required.

## 1 INTRODUCTION

Soft tissue sarcomas (STS) are a heterogeneous collection of rare tumors of mesenchymal origin which represent less than 1% of adult cancers.^1^ Management of STS is challenging due to their wide variety of histological sub‐types with differing clinical, phenotypical, and genomic characteristics that impact their sensitivity to treatment.^2^ Primary cardiac sarcoma (PCS) is an extremely rare subset of STS, with an estimated incidence of 0.001%‐0.03%.^3^, ^4^, ^5^ It is distinguished from a diverse and more common group of other cardiac tumors, including benign tumors (ie, myxomas), and secondary neoplasms metastasizing to the heart (eg, lung, breast, renal, melanoma), by arising from pluripotent mesenchymal cells within the heart.^6^

Patients typically present with symptoms related to the local effects of the tumor, which can include chest pain, arrhythmias, peripheral edema, dyspnea, orthopnea, congestive heart failure, and pericardial tamponade. All four cardiac chambers may be involved, as well as the myocardium and pericardium, with certain tumor types having a predilection to specific sites. The overall prognosis of patients with primary cardiac sarcoma is poor, with median overall survival (OS) ranging from 9 to 27 months in recent case series.^7^, ^8^, ^9^, ^10^, ^11^, ^12^, ^13^, ^14^

Due to the rarity of this disease, there is limited evidence supporting specific therapies. An important prognostic indicator is resectability of disease with studies documenting a median OS of 38 months in patients with completely resected disease vs 11 months in those that were unresectable.^14^ The use of adjuvant chemotherapy^12^, ^13^ or radiotherapy^14^ may improve outcomes; however, studies are limited by small sample sizes and lack of randomization. For those with unresectable disease, chemotherapy is the mainstay of treatment but survival is typically poor. Given the limited information guiding treatment choices in PCS, we aimed to assess outcomes in a contemporary cohort across six multi‐national cancer centers and identify the prognostic impact of clinico‐pathological variables.

## 2 METHODS

### 2.1 Patient selection

Medical records from patients diagnosed with PCS between 1 January 1995 and 31 December 2015 were retrospectively collected from six institutions across three continents (Princess Margaret Cancer Centre, Canada; Mount Sinai Hospital, Canada; British Columbia Cancer Agency, Canada; National Taiwan University Hospital, Taiwan; Prince of Wales Hospital, Hong Kong; Rabin Medical Center, Israel). Patients were excluded if the site of origin was not cardiac (ie. cardiac metastasis), and the pathology was not a sarcoma histology. Metastatic disease from PCS at the time of diagnosis was not an exclusion criterion. This study was approved by the institutional research ethics committee in each participating institution respectively.

Clinical variables collected included age, gender, date of diagnosis, histological subgroup, treatment modality received, date of progression, and survival. Tumor size was measured by the maximal length of the primary tumor on the imaging of either computed imaging (CT) or magnetic resonance imaging (MRI).

### 2.2 Statistical analysis

Descriptive statistics were used to report demographic and clinical data and presented as means, medians, and ranges for continuous factors and frequencies for categorical factors. Progression‐free survival (PFS) was measured from the time of diagnosis until disease progression or death from any cause. OS was measured from the time of diagnosis until death from any cause. PFS and OS were estimated using the Kaplan‐Meier product limit method. Differences in survival curves between groups were estimated using the log‐rank test. The relations of all prognostic factors to OS were evaluated by univariate Cox proportional hazards regression models. Clinical variables with a *P*‐value <0.15 were selected into the final multi‐variate models. We defined long‐term survivors as those who survived longer than 36 months (double the duration of median OS). All statistical analyses were performed using SAS 9.4 software (SAS Institute Inc NC, USA). A *P*‐value of <0.05 was considered statistically significant.

## 3 RESULTS

A total of 61 patients with PCS were identified. The median age at diagnosis was 46 (range 18‐79) with a median tumor size of 50 mm (range 19‐84 mm). PCS equally originated from left (n = 30) and right (n = 30) sides of the heart with one patients’ tumor originating from the pericardium. The most common histological subgroups were angiosarcoma (N = 24, 39.3%), sarcoma NOS (n = 14, 23.0%), and intimal sarcoma (n = 8, 13.1%). A summary of clinico‐pathological features is shown in Table 1.

**Table 1:** Clinico‐pathological characteristics of the cohort

	N = 61	%
Age		
Median [min‐max]	46 [18, 79]	
Gender		
Male	28	45.9
Female	33	54.1
Tumor size (mm)		
Median[min‐max]	50 [19, 84]	
Initial stage at diagnosis		
Local	39	63.9
Metastatic	22	36.1
Histology		
Angiosarcoma	24	39.3
Sarcoma, NOS	14	23.0
Intimal sarcoma	8	13.1
Leiomyosarcoma	4	6.6
Myxofibrosarcoma	2	3.3
Pleomorphic sarcoma	4	6.6
Synovial sarcoma	2	3.3
Liposarcoma	2	3.3
Rhabdomyosarcoma	1	1.6
Primary tumor location		
Right heart	30	49.2
Left heart	30	49.2
Pericardium	1	1.6
Surgery to the primary tumor		
Yes	46	75.4
No	15	24.6
Type of resection		
Clear margin (R0)	5	8.2
Microscopic (R1)	11	18.0
Macroscopic (R2)	27	44.2
Unknown	3	4.9
Tumor grade		
Low/Intermediate	6	13.9
High	37	86.1
Unknown	18	29.5

NOS, Not otherwise specified.

At diagnosis, more patients had localized disease (n = 39, 63.9%) than metastatic disease (n = 22, 36.1%). Overall, 46 patients (75%; localized disease: 37/39 (95%); metastatic disease: 9/22 (41%)) received surgery to the primary cardiac tumor, but only 5 (8%) patients achieved R0 resection. For patients presenting with localized disease, multi‐modality treatment (MMT) was given to 23 patients (59%; surgery and chemotherapy (n = 16, 70%); surgery and radiation (n = 2, 8%); surgery, radiation, and chemotherapy (n = 5, 22%)). In metastatic disease patients, MMT was provided to five (23%) patients with all receiving surgery and chemotherapy treatment, and no radiotherapy to the primary tumor. Eleven patients (50%) received chemotherapy as the only treatment, and five patients (23%) received palliative care only after the initial diagnosis of metastatic disease. Overall, patients initially diagnosed with metastatic disease were less likely to receive MMT (odds ratio = 0.20, [95% confidence interval (CI) = 0.06‐0.67], *P* < 0.01).

### 3.1 Survival analysis

**Figure 1:**
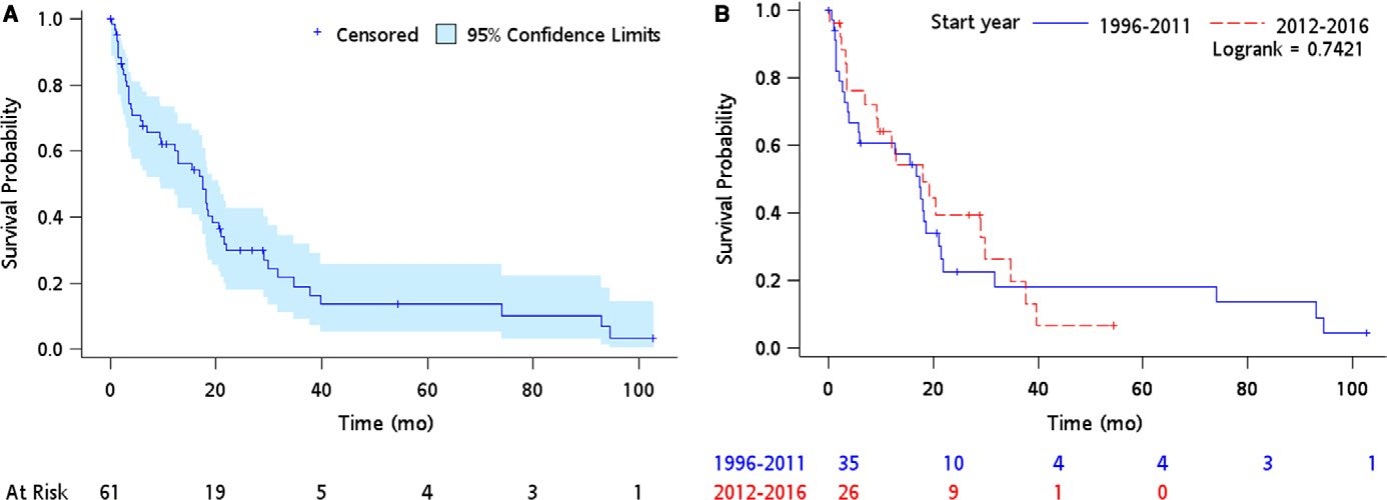
The overall survival curves of (A) the whole cohort (B) cohorts based on the year of diagnosis

After a median follow‐up of 54.4 months, 47 events were observed. The median OS was 17.5 months (95% CI 9.5‐20.6 months) (Figure 1A). The median OS for those with R0, R1/R2 and who had not received cardiac surgery was 34.8, 18.3, and 8.9 months, respectively (*P* < 0.01). To evaluate if the prognosis of PCS has improved in a contemporary group, an OS analysis was performed to compare those diagnosed in the past 5 years (2012‐2016) compared with a prior cohort (1996‐2011). There were no differences in the OS between the two cohorts (1996‐2011:17.5 months; 2012‐2016:18.1 months, *P* = NS, Figure 1B).

We then aimed to determine prognostic factors for survival. In the univariate analysis, three clinical variables were significant prognostic factors: age ≥65 (hazard ratio (HR) = 2.92 [1.27‐6.73], *P* = 0.01), metastatic disease at diagnosis (HR = 2.09 [1.09‐4.00], *P* = 0.03), and receiving surgery to primary tumor (HR = 0.41 [0.19‐0.85], *P* = 0.02) (Table 2). In addition, there was a trend to significance in the use of MMT (HR = 0.60 [0.34‐1.08], *P* = 0.09) and in angiosarcoma vs other histological subgroup (HR = 1.84 [0.94‐3.6], *P* = 0.07). Using univariate analysis selection criteria, we entered limited clinical variables (age, stage, histology, PCS location, surgery, and MMT) into a multi‐variate analysis model. In this analysis, only younger age remained as a significant favorable prognostic factor (*P* = 0.01) whilst localized diagnostic stage had a trend toward significant favorable prognostic impact (*P* = 0.08)(Table 2).

**Table 2:** Prognostic impact clinico‐pathological variables based on uni‐ and multi‐variate Cox proportional hazard models

	Univariate HR (95% CI)	Univariate *P*‐value	Multi‐variate HR (95% CI)	Multi‐variate *P*‐value
Age (≥65 vs <65)	2.92 (1.27, 6.73)	0.01	3.54 (1.35, 9.26)	0.01
Gender (female vs male)	1.05 (0.59, 1.87)	0.86		
Tumor size (mm) (≥49 vs <49)	1.14 (0.60, 2.17)	0.70		
Initial stage at diagnosis (metastatic vs localized)	2.09 (1.09, 4.00)	0.03	2.13 (0.91, 4.96)	0.08
Histology (angiosarcoma vs others)	1.84 (0.94, 3.60)	0.07	0.93 (0.37, 2.36)	0.89
Primary tumor location (left vs right side)	0.53 (0.28, 1.00)	0.05	0.66 (0.27, 1.60)	0.36
Tumor grade (high vs low/intermediate)	2.03 (0.71, 5.83)	0.19		
Surgery to primary tumor (yes vs no)	0.41 (0.19, 0.85)	0.02	0.93 (0.35, 2.46)	0.89
Type of resection (R1+R2 vs R0)	2.37 (0.71, 7.91)	0.16		
Multi‐modality treatment (yes vs. single treatment)	0.60 (0.34, 1.08)	0.09	0.72 (0.35, 1.48)	0.38

HR, Hazard ratio; RT, radiotherapy.

### 3.2 Advanced disease

Forty‐eight patients with PCS either presented with metastatic disease or had subsequent relapse after localized disease. The most common metastatic sites were lung (n = 22, 46%) and bone (n = 8, 17%). Eleven (23%) patients had cardiac or pericardial recurrences with two receiving prior cardiac radiation. First‐line treatment for patients presenting with advanced disease was chemotherapy in 30 (63%), radiotherapy in 7 (15%), and surgery in 5 (10%). Five patients received only palliative care and three patients were lost to follow‐up.

**Figure 2:**
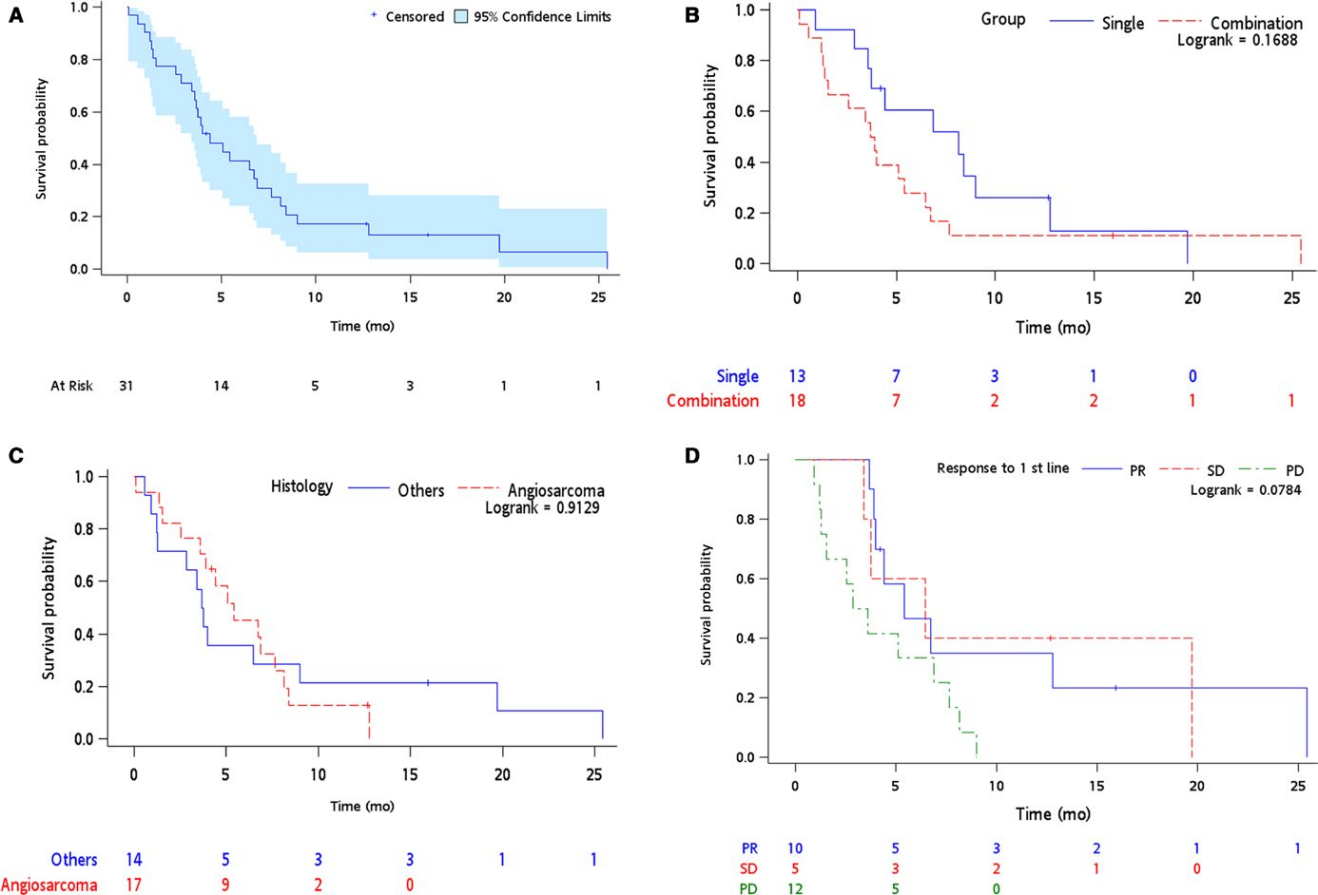
Progression‐free survival Kaplan‐Meier curves in advanced patients (A) all patients received first‐line chemotherapy (B) single vs combination regimen (C) angiosarcoma vs other histologies (D) based on response to first‐line chemotherapy

Thirty‐one (65%) advanced disease patients received at least one line of systemic chemotherapy (median: one line of treatment, range 1‐4) with 18 (58%) and 13 (42%) patients receiving combination and single‐agent regimens, respectively. The overall best response (complete (CR) or partial response (PR)) to first‐line palliative chemotherapy was 32% (n = 10), stable disease 16% (n = 5), disease progression in 39% (n = 12), and not evaluable in 13% (n = 4). Of the 10 patients with response (CR = 1; PR = 9), 8 (80%) patients had treatment with anthracycline‐containing regimen and 7 (70%) had angiosarcoma histology (Table 3). The median PFS of first‐line palliative chemotherapy was 4.4 months [95% CI 2.86‐7.67 months]. In univariate analysis, combination vs single agent, angiosarcoma vs other histologies, or a response to first‐line treatment was not significantly associated with improved PFS (Figure 2). Newer available options for STS such as pazopanib and trabectedin were utilized in five and one patients respectively beyond first‐line treatment, although none were evaluable for response.

**Table 3:** Chemotherapy regimens associated with response in the first‐line chemotherapy

Regimen	Patient number (total = 10)
Single‐agent doxorubicin	2
Single‐agent liposomal doxorubicin	2
Doxorubicin + ifosfamide	3
Liposomal doxorubicin plus paclitaxel	1
Bevacizumab + ifosfamide + etoposide	1
Ifosfamide + etoposide	1

### 3.3 Long‐term survivors

There were seven (11%) patients surviving longer than 36 months (range 38‐103 months), and two patients were alive at the time of follow‐up. All seven patients were under age 65 (median: 41 years old, range 22‐57) and initially diagnosed with localized disease (intimal sarcoma (n = 2), sarcoma NOS (n = 3), myxofibrosarcoma (n = 1), leiomyosarcoma (n = 1)). Surgery was a line of treatment to all seven patients but only one patient had a R0 resection. MMT were provided to four patients (surgery + adjuvant chemotherapy (n = 3), surgery +adjuvant chemotherapy +radiotherapy (n = 1)). Six of the seven long‐term survivors eventually relapsed, with four of these relapses occurring locally whilst two patients had first relapse outside of the cardiac area.

## 4 DISCUSSION

The optimal management of patients with PCS is unclear given that the evidence base is limited to small retrospective series. Although the primary aim is complete resection, most patients develop recurrent disease despite surgical resection and survival remains poor. In this study, we aimed to assess outcomes in a contemporary cohort across six multi‐national cancer centers and identify the prognostic impact of clinico‐pathological variables. Our major findings were that younger age, local stage, and resection of the primary tumor were independent favorable prognostic factors. Potentially due to sample size, only younger age remained a significant favorable prognostic factor on multi‐variate analysis. Contrary to other groups,^11^ little improvement was noted over the 20‐year period. The exact reason for this is unclear and highlights the need for dedicated evaluation in the modern era of treatment strategies.

The median OS of17.5 months with a 5‐year survival of less than 20% (Figure 1) is consistent with other multi‐institutional reports of PCS and worse than expected outcomes in extremity/visceral based STS. Of note, our cohort had a higher rate of de novo metastatic disease (36%) compared to other similar reports,^11^, ^14^ which may have affected our survival rates. Histological breakdown was similar to previous publications with a predominance of sarcoma NOS and angiosarcoma.^14^, ^15^ Although rhabdomyosarcoma has been reported to occur in up to 20% of all PCS, only one case was identified in this cohort.^16^ The rate of sarcoma NOS (23%) highlights work that will need to be undertaken including refining pathological review with implementation of advances in morphologic criteria, immunostaining for histologic differentiation, and molecular assays.^17^

There was a trend for unfavorable prognostic effect of right‐ vs left‐sided tumors. Regardless of location, attempting surgical resection remains important for management of this disease and may be associated with a survival advantage.^14^ In patients presenting with localized disease, only five of 43 patients had R0 resections reflecting the difficulty of obtaining clear surgical margins or that PCS may only become apparent in retrospect on pathological evaluation. Nevertheless, surgery, whether complete or incomplete, was a prognostic factor for survival with a median OS for those with R0, or R1/R2 of 34.8 and 18.3 months as opposed to 8.9 months for those with unresectable disease (*P* < 0.01). In addition, we report long‐term survivors in our series, particularly for those in whom resections were attempted. Thus, it is possible that a paradigm shift may be required, distinct from the prevailing management of extremity based STS, where even incomplete resection may be of benefit for patients with PCS.^14^ In our cohort, 11% of individuals were alive for longer than 36 months of which surgery was provided to all albeit with only a single patient having an R0 resection. Of note, other groups have reported more prolonged survival in those with complete resection.^9^, ^18^, ^19^ For example, in the French Sarcoma group, the median OS was 39 months after R0 resection vs 18 months for R1/R2 and 11 months in non‐resected patients.^14^

Because the heart is such a critical organ, control of the primary site may be an important component for patients with both localized and metastatic PCS. Multi‐modality treatment (MMT) was given to 23 and five patients with localized (59%) and metastatic (23%) disease respectively, primarily with surgery and adjuvant chemotherapy, and there was a trend to improved survival in those who received MMT (HR 0.60, *P* = 0.09). In the Cleveland group, MMT was associated with improved outcome with median survival of 36.5 months compared to 14.1 months treated with single modality.^11^ Given the rarity of this subtype and the retrospective nature of this study, the role of MMT should be applied cautiously and discussed with clinicians with experience of PCS in both limited and advanced disease. For patients with advanced disease, we identified a response rate to first‐line chemotherapy of 32% typically utilizing an anthracycline‐based approach. Nevertheless, the responses were not durable with a median PFS of 4.4 months. Future evaluation aimed at investigating the activity of newer systemic treatments, including but not limited to biologics and immunotherapeutic approaches, would be beneficial. Surprisingly, no responses to taxanes were seen in patients with angiosarcoma subtypes in our study cohort. This is in contrast to favorable responses to taxanes in non‐cardiac angiosarcomas reported in several prior series.^20^, ^21^

Understanding the molecular characteristics will be important in understanding the unique biology of PCS and may allow identification of molecularly targeted agents. Preliminary investigation in 70 cases of PCS that underwent molecular analysis demonstrated potentially actionable aberrations including amplification of*MDM2* and *PDGFRA*.^22^ This is supported by preclinical work in intimal sarcoma (a subtype of sarcoma akin to PCS arising in large arteries) where *PDGFRA *amplifications are common, together with activation of *EGFR* and *MDM2. *These mutations *s*upport the investigation of receptor tyrosine kinases blocking downstream pathways.^23^, ^24^

There are clear limitations of this study. Firstly, this is a retrospective review across multiple international institutions with variation in sarcoma practices and accessibility to certain systemic therapies as well as the intensity and schedule of the systemic treatment, thus it is unknown whether our findings can be translated to other centers managing PCS. The side effect profile of systemic treatments (including both anthracycline‐based regimen and non‐anthracycline‐based regimens) were not extracted from the medical chart of each hospital, limiting the ability of our study to provide these details in the clinical care of PCS patients. In addition, we did not capture individuals who underwent aggressive surgical techniques such as transplantation or cardiac auto‐transplantation thus cannot speculate of the potential benefit of these approaches.^25^ Lastly, we did not conduct central pathology review although all cases were reported by expert sarcoma pathologists at each center.

## 5 SUMMARY

We identified that younger age at diagnosis, localized disease, and surgical management to primary tumor was associated with improved outcome for patients with PCS. No significant improvement in OS was identified in patients presenting throughout the 20‐year period of this review. Given the small numbers at any center and overall poor prognosis of PCS, dedicated, planned, multicenter studies are required for this rare sarcoma subtype.

## CONFLICT OF INTEREST

All authors declared no conflict of interests.
